# Lichen Secondary Metabolite, Physciosporin, Inhibits Lung Cancer Cell Motility

**DOI:** 10.1371/journal.pone.0137889

**Published:** 2015-09-15

**Authors:** Yi Yang, So-Yeon Park, Thanh Thi Nguyen, Young Hyun Yu, Tru Van Nguyen, Eun Gene Sun, Jayalal Udeni, Min-Hye Jeong, Iris Pereira, Cheol Moon, Hyung-Ho Ha, Kyung Keun Kim, Jae-Seoun Hur, Hangun Kim

**Affiliations:** 1 Korean Lichen Research Institute, Sunchon National University, Sunchon 540–950, Republic of Korea; 2 College of Pharmacy and Research Institute of Life and Pharmaceutical Sciences, Sunchon National University, Sunchon 540–950, Republic of Korea; 3 Faculty of Natural Science and Technology, Tay Nguyen University, Buon Ma Thuot, Vietnam; 4 Medical Research Center for Gene Regulation, Chonnam National University Medical School, Gwangju 500–872, Republic of Korea; 5 Institute of Biological Sciences, Universidad de Talca, Talca 747–721, Chile; Peking University Cancer Hospital & Institute, CHINA

## Abstract

Lichens produce various unique chemicals that can be used for pharmaceutical purposes. To screen for novel lichen secondary metabolites showing inhibitory activity against lung cancer cell motility, we tested acetone extracts of 13 lichen samples collected in Chile. Physciosporin, isolated from *Pseudocyphellaria coriacea* (Hook f. & Taylor) D.J. Galloway & P. James, was identified as an effective compound and showed significant inhibitory activity in migration and invasion assays against human lung cancer cells. Physciosporin treatment reduced both protein and mRNA levels of N-cadherin with concomitant decreases in the levels of epithelial-mesenchymal transition markers such as snail and twist. Physciosporin also suppressed KITENIN (KAI1 C-terminal interacting tetraspanin)-mediated AP-1 activity in both the absence and presence of epidermal growth factor stimulation. Quantitative real-time PCR analysis showed that the expression of the metastasis suppressor gene, KAI1, was increased while that of the metastasis enhancer gene, KITENIN, was dramatically decreased by physciosporin. Particularly, the activity of 3’-untranslated region of KITENIN was decreased by physciosporin. Moreover, Cdc42 and Rac1 activities were decreased by physciosporin. These results demonstrated that the lichen secondary metabolite, physciosporin, inhibits lung cancer cell motility through novel mechanisms of action.

## Introduction

Lung cancer is the most common cancer and the leading cause of cancer-related death in humans worldwide [1, 2]. There were about 1.8 million new cases of lung cancer diagnosed in 2012, accounting for 12.9% of the total cases of cancer [1, 2]. Due to the lack of efficient treatment for advanced disease, the prognosis of lung cancer is still poor, with less than 15% surviving 5 years after diagnosis [3]. When lung cancer is diagnosed at presentation with symptoms, it carries an extremely poor prognosis, with an overall 5-year survival of 16% in the USA and less than 10% in the UK [4]. In lung cancer, metastasis is the leading cause of death, and unraveling the mechanism of tumor progression and metastasis is thus of great importance [5]. In the initial steps of local invasion, the signaling pathways that control the cytoskeletal dynamics of tumor cells, the turnover of cell–matrix and the cell–cell junctions were activated [6]. Therefore, development of new chemical agents that inhibit cancer cell migration and invasion by targeting the above signal is required in treatment of advanced cancers.

Lichens produce diverse secondary metabolites that show a variety of biological activities including anti-cancer activity [7]. To date, nearly a thousand secondary metabolites of lichens have been discovered and some of these unique lichen metabolites are effective against various *in vitro* cancer models [8]. However, although lichens are a source for screening for anti-cancer active compounds, only a small number of compounds have been tested [9]. Therefore, the current study examined the inhibitory activity of 13 Chilean lichen species against migration and invasion ability of human lung cancer cells and further investigated the possible molecular mechanisms underlying their anti-metastatic activity to identify potential novel anti-metastasis agents.

## Materials and Methods

### Preparation of lichen extracts

Thalli of the lichens were collected from Chile in January of 2009 and 2012 during field trips in the National Park of Torres Del Paine, Patagonia, organized by Dr. Pereira at Talca University, Talca, Chile. The permit to collect lichen specimens from location was issued by the Administration of the National Forestry Corporation (CONAF) of Punta Arenas and the Administration of the National Park Torres del Paine, Magallanes region and Chilean Antarctic, which is part of the National System of Protected Wild Areas of the State of Chile. The field studies did not involve any endangered or protected species. The duplicates were deposited at the Korean Lichen and Allied Bioresource Center (KOLABIC) in the Korean Lichen Research Institute (KoLRI), Sunchon National University, Korea.

### Thin Layer Chromatography (TLC) analysis of lichen material

Lichen thalli were soaked in 1 mL acetone for 5 minutes in 1.5 mL EP tubes, and the concentrated solution was spotted on Silica gel 60 F254 pre-coated plates (Merck Millipore, Darmstadt, Germany) using a microcap several times. Solvent A [Toluene: Dioxin: Acetic acid 180: 45: 5 (v/v/v)], described in Culberson’s improved standardized method [10], was used in this study. The TLC plate spotted with samples was loaded in a twin trough chamber pre-saturated with solvent system A and was removed from the chamber when the solvent front reached 14cm from the starting baseline. The results were visualized by examination and marking in daylight (for pigments) and under UV at 254 and 350 nm. Following this, the plates were sprayed with 10% aqueous sulfuric acid and heated at 110°C to ensure full visualization. After charring, the specific colors of substances, both in daylight and under UV (350nm), were noted.

Two lichen species *Lethariella cladonioides (Nyl*.*) Krog* (Atranorin) and *Usnea longissimi* (usnic acid) were used as the standard control. Based on the standardized method [10, 11], relative R_f_ value was determined to help identify each spot [12].

### Separation and identification of physciosporin

*Pseudocyphellaria coriacea* (CL090002) extract was separated by TLC as described above. The spot identified as physciosporin was scratched off and eluted with acetone. The supernatant was dried and weighed after centrifugation. High-performance liquid chromatography (HPLC) assay was performed to confirm single peak within the purified sample, and, then, structure of physciosporin was confirmed by nuclear magnetic resonance (NMR) analysis (Figs A and B in S1 File).

### Cell culture

The human lung cancer cells including A549, H1650 and H1975 were used in this study. Cells were cultured in RPMI 1640 culture medium supplemented with 10% fetal bovine serum, 1% Penicillin-Streptomycin solution under a humidified 5% CO_2_ atmosphere at 37°C in an incubator.

### Wound healing assay

A549 cells were plated at a density of 2.5~3 × 10^5^ cells/well on 6-well tissue culture plates (Corning, New York, USA) and grown overnight to confluence. Monolayer cells were scratched with a sterile pipette tip to create a wound. The cells were then washed twice with serum-free RPMI 1640 to remove floating cells and incubated in RPMI1640 culture medium supplemented with 2% FBS with 5 μg/mL of acetone extract of *P*. *coriacea* or physciosporin. Photographs of cells were taken at 0, 24, 48, and 72 h after wounding to measure the width of the wound. For each sample, an average of five wound assays was taken to determine the average rate of migration. Experiments were repeated at least three times.

### Invasion assay

Invasion assays were performed in Boyden chambers (Corning, New York, USA). The polycarbonate filters (8 μm pore size, Corning) pre-coated with 1% gelatin were used for invasion assays. Cells (2×10^6^) in 120 μL of medium (RPMI 1640 containing 0.2% BSA dissolved in PBS) with or without 5 μg/mL crude lichen extracts or physciosporin were seeded in the upper chamber. Then 400 μL medium with 10 μg/mL fibronectin was added to the lower chamber to serve as a chemotactic agent. After 24 h incubation, the cells in the upper chamber were fixed with Diff Quik kit (Sysmex). Then the cells attached in the upper chamber were mechanically removed by cotton swab. The cells adhering to the under-side of the filter were stained and counted under upright microscope (5 fields/chamber). Each invasion assay was repeated in three independent experiments.

### Western blotting

Cells treated with 5 μg/mL of acetone extract of *P*. *coriacea* or physciosporin for 24 h were washed twice with ice-cold PBS and lysed in lysis buffer [13]. The antibodies used were purchased from Cell Signaling Technology (N-cadherin, α-tubulin) and BD Biosciences (E-cadherin). Antibodies were detected with horseradish peroxidase-conjugated secondary antibody (Thermo) using Immobilon Western Chemiluminescent HRP Substrate Kit (MILLIPORE) and luminescence imaging (Image Quant LAS 4000 mini). Bands were measured by Multi-Gauge 3.0 and their relative density calculated based on the density of the α-tubulin bands in each sample. Values were expressed as arbitrary densitometric units corresponding to signal intensity.

### Quantitative RT-PCR

The quantitative RT-PCR (qRT-PCR) was performed as previously described [14]. Briefly, total RNA was isolated from human lung cancer cells by using RNAiso Plus (TaKaRa, Otsu, Shiga Shiga 520–2193, Japan) according to the manufacturer’s instructions. Total RNA from each group of treated cells was converted to cDNA using a M-MLV reverse Transcriptase kit (Invitrogen, Carlsbad, USA) and SYBR green (Enzynomics, Seoul, Korea). The primers used for real-time PCR were Snail (forward) 5'-tcccgggcaatttaacaatg-3' and (reverse) 5'-tgggagacacatcggtcga-3'; Twist (forward) 5'-cgggagtccgcagtctta-3' and (reverse) 5'-tgaatcttgctcagcttgtc-3'; N-cadherin (forward) 5'-ctcctatgagtggaacaggaacg-3' and (reverse) 5'-ttggatcaatgtcataatcaagtgctgta-3'; KAI1 (forward) 5'-gctcatgggcttgggct-3' and (reverse) 5'-gagctcagtcacgatgccgc-3'; KITENIN (forward) 5’-cggaataaagacggcagagg-3’ and (reverse) 5’-tgctccgaggtgcctgtgat-3’; GAPDH (forward) 5’-atcaccatcttccaggagcga-3’ and (reverse) 5’-agttgtcatggatgaccttggc-3’. qRT-PCR reactions and analyses were performed using CFX (Bio-Rad, Hercules, USA).

### Reporter assay

For AP-1 reporter assay, HEK293T cells were transfected with KITENIN expression plasmid and AP1 reporter plasmid. After 12 h transfection, cells were treated with 5 μg/mL of acetone extracts of *P*. *coriacea* or physciosporin for 48h with or without EGF and then analyzed using a Dual-Luciferase® reporter assay system (Promega, Madison, WI, USA). The Renilla luciferase reporter plasmid (pRL-TK) was used as an internal control for the transfection efficiency. The activity for TOPFLASH reporter, NF-κb and STAT reporter were also tested in our study.

For KITENIN promoter reporter assay, human KITENIN promoter (-2344 to + 536) was amplified by PCR from genomic DNA of HEK293T cells using primers designed from the genomic sequence of the KITENIN locus (1p13.1). The PCR products were digested with *Kpn*I/*Bgl*II and cloned into the pGL3basic plasmid (Promega, Madison, WI, USA) upstream of the luciferase reporter gene. Construction was confirmed by sequencing. The KITENIN 3’-untranlated region (3’-UTR) assay was performed as previously described [15]. The experiments were performed in triplicate, and at least three results from independent experiments were included in an analysis. Fold changes were calculated using values normalized to Renilla luciferase activity.

### Affinity-precipitation of cellular GTPases

The cellular Rac1 and Cdc42 activities were determined using GST-RBD/PBD as previously described [16]. Briefly, the cells were lysed in lysis buffer. The lysates were incubated with GST- RBD/PBD beads at RT for 1 h. The beads were then washed four times with washing buffer. The bound Rac1 and Cdc42 proteins were detected by immunoblotting using a monoclonal antibody against Rac1 (MILLIPORE 05–389) and Cdc42 (SANTA CRUZ SC-87). The relative activity of each GTPase was determined by quantifying each band of GTP-bound GTPase and the total amount of GTPase using Multi-Gauge 3.0, and the values of the GTP-bound bands were normalized to that of the total amount. All results were determined using three different exposures from at least three independent experiments.

## Results

### Acetone extracts of lichens collected in Chile inhibit A549 cell motility

**Fig 1:**
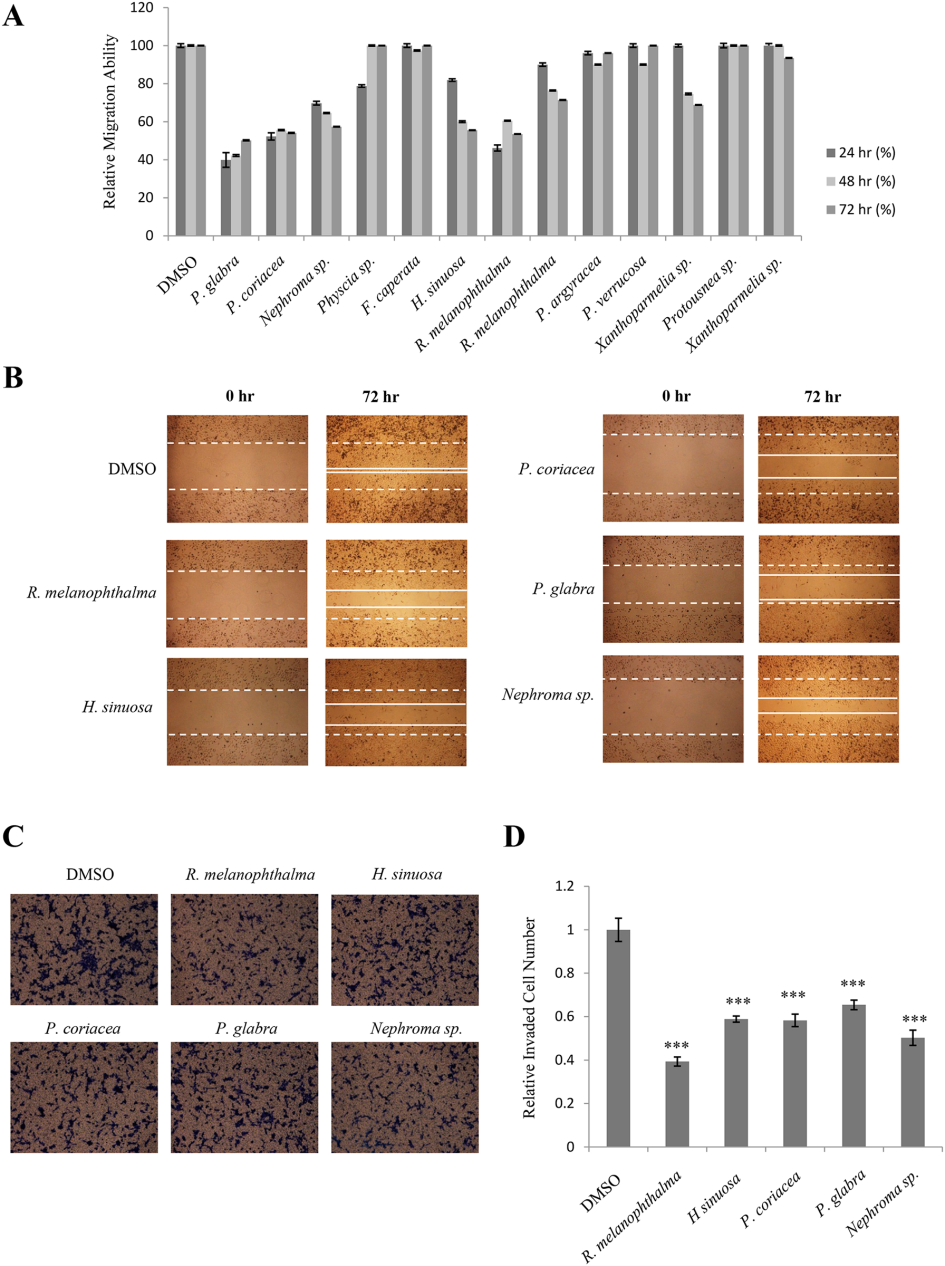
Acetone extracts of lichens collected in Chile inhibited A549 cell motility. (A) Quantitative analysis of migration assays of A549 cells treated with 5 μg/mL of acetone extracts of *Pseudocyphellaria glabra*, *Pseudocyphellaria coriacea*, *Nephroma* sp., *Physcia* sp., *Flavoparmelia caperata*, *Hypotrachyna sinuosa*, *Rhizoplaca melanophthalma*, *Rhizoplaca melanophthalma*, *Pseudocyphellaria argyracea*, *Pseudocyphellaria verrucosa*, *Xanthoparmelia* sp., *Protousnea sp*. and *Xanthoparmelia* sp.. (B) Representative images of migration assays of A549 cells treated with the extracts of *R*. *melanophthalma*, *H*. *sinuosa*, *P*. *coriacea*, *P*. *glabra* and *Nephroma* sp. (C-D) Invasion assays of A549 cells treated with 5 μg/mL of acetone extracts of *R*. *melanophthalma*, *H*. *sinuosa*, *P*. *coriacea*, *P*. *glabra and Nephroma sp*. (C) and quantitative analysis of invaded cell numbers in each treatment (D). Quantitative data were obtained from three independent experiments, n = 3. Data represent mean ± S.E.M. (standard error of the mean). ***p<0.001 compared to DMSO-treated A549 cells.

Invasion and migration play a crucial role in the metastasis of cancer cells. To find an inhibitory substance from lichen secondary metabolites, wound healing assays were performed in A549 human lung cancer cells as an initial screening for acetone extracts of 13 Chilean lichens (Table 1) [17–20]. As shown in Fig 1A, *Rhizoplaca melanophthalma*, *Hypotrachyna sinuosa*, *Pseudocyphellaria coriacea*, *Pseudocyphellaria glabra* and *Nephroma sp*. inhibited the migration of A549 cells at a concentration of 5 μg/mL. This concentration was used as no cytotoxicity was observed at this concentration (data not shown). The distances between the edges of the wounds for cells treated with the five lichen extracts were wider than those for DMSO-treated cells (Fig 1B).

**Table 1:** Thirteen Chile lichen species used in this study.

Collection No.	Family	Species name	Known lichen substances	Reference
CL090001	*Lobariaceae*	*Pseudocyphellaria glabra*	7β-acetoxyhopan-22-ol hopane-15α,22-diol, stictic and constictic acid	[17]
CL090002	*Lobariaceae*	*Pseudocyphellaria coriacea*	7β-acetoxyhopan-22-ol hopane-7β,22-diol, 7β-acetoxyhopan-22-ol	[17]
CL090003	*Nephromataceae*	*Nephroma* sp.	Zeorin	[18]
CL090006	*Physciaceae*	*Physcia* sp.	Atranorin, zeorin	[18]
CL120051	*Parmeliaceae*	*Flavoparmelia caperata*	Usnic,ptorocetraric,caperatic acid and atranorin	[18]
CL120055	*Parmeliaceae*	*Hypotrachyna sinuosa*	Usnic acid salazinic acid	[18]
CL120113	*Lecanoraceae*	*Rhizoplaca melanophthalma*	Usnic acid	[18]
CL120124	*Lecanoraceae*	*Rhizoplaca melanophthalma*	Usnic acid	[18]
CL120181	*Lobariaceae*	*Pseudocyphellaria argyracea*	7β-acetoxyhopan-22-ol hopane-7β,22-diol, 7β-acetoxyhopan-22-ol,methyl gyrophorate and gyrophoric acid	[17]
CL120206	*Lobariaceae*	*Pseudocyphellaria verrucosa*	Gyrophoric acid	[19]
CL120330	*Parmeliaceae*	*Xanthoparmelia* sp.	Usnic, salazinic,norstic,stictic,,barbatic and diffractaic acid	[18]
CL120340	*Parmeliaceae*	*Protousnea* sp.	Sekikaic and divaricatic acid	[20]
CL120371	*Parmeliaceae*	*Xanthoparmelia* sp.	Usnic, salazinic,norstic,stictic,,barbatic and diffractaic acid	[18]

To further check whether the above lichen extracts had inhibitory activity against invasion of A549 cells, invasion assays were performed using gelatin-coated two-well chambers. As shown in Fig 1C, cells treated with the acetone extracts of the five lichens showed fewer numbers of invaded cells when compared to DMSO-treated control. Quantitative analysis of the data revealed that the differences were significant (Fig 1D). These results indicate that acetone extracts of *R*. *melanophthalma*, *H*. *sinuosa*, *P*. *coriacea*, *P*. *glabra* and *Nephroma sp*. exhibit inhibitory activity against A549 cell motility.

### Physciosporin was identified as an active lichen secondary metabolite from *P*. *coriacea* in the inhibition of lung cancer cell motility

**Fig 2:**
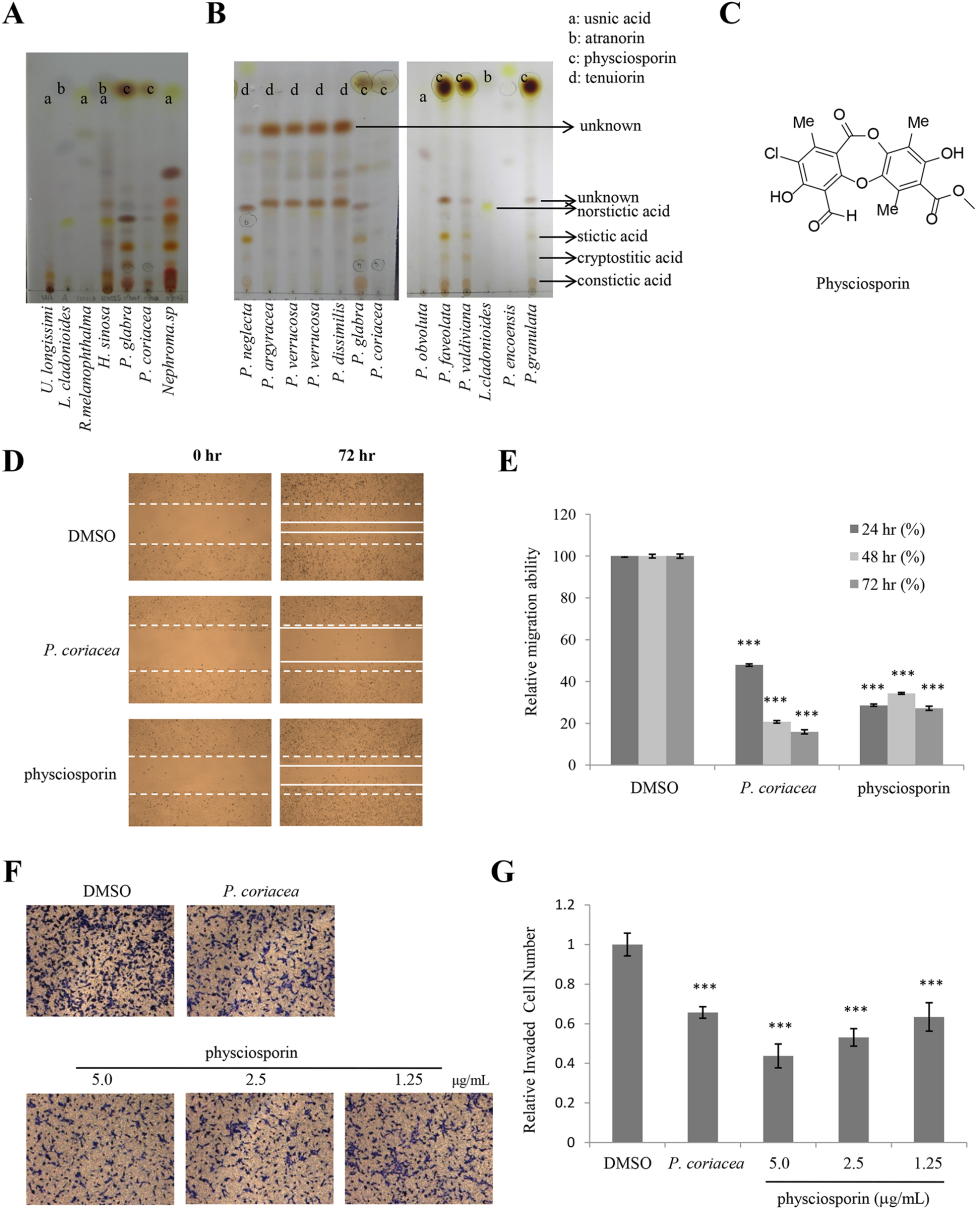
Physciosporin was identified as an active lichen secondary metabolite from *P*. *coriacea* in the inhibition of lung cancer cell motility. (A-B) Thin Layer Chromatography (TLC) analysis using (Toluene: Dioxin: Acetic acid = 180: 45: 5, v/v/v) solvent system for lichen extracts having inhibitory activity in A549 cell motility (A), the lichens in *Pseudocyphellaria* genus (B). ‘a’ denotes location of spot for usnic acid; ‘b’, for atranorin; ‘c’, for physciosporin; ‘d’, for tenuiorin. Lichen species *L*. *cladonioides* and *U*. *longissimi* were used as standard control for atranorin and usnic acid, respectively. (C) Chemical structure of physciosporin. (D-E) Migration assay of A549 cells treated with 5 μg/mL of acetone extract of *P*. *coriacea* or 5 μg/mL physciosporin (D), and quantitative analysis of wound length (E). (F–G) Invasion assays of A549 cells treated with 5 μg/mL of acetone extract of *P*. *coriacea* or physciosporin (F), and quantitative analysis of invaded cell numbers in each treatment (G). Quantitative data were obtained from three independent experiments, n = 3. Data represent mean ± S.E.M. (standard error of the mean). ***p<0.001 compared to DMSO-treated A549 cells.

To investigate the main compound of these five candidate lichens, five lichen acetone extracts were individually analyzed by TLC (Fig 2A). Based on the relative R_f_ values [12], *R*. *melanophthalma*, *H*. *sinuosa*, *and Nephroma sp*. share the same major component, usnic acid (spot a, Fig 2A), while *P*. *glabra* and *P*. *coriacea* have distinct main compounds (spot c, Fig 2A). As usnic acid is the most extensively studied lichen metabolite for anti-cancer activity, we chose ‘spot c’ for further study. ‘Spot c’ was distinctively observed within *P*. *glabra* and *P*. *coriacea* among the other *Pseudocyphellaria* genus that possesses tenuiorin (spot d) and other unknown metabolites as the main compound (left panel, Fig 2B). As ‘spot c’ in *P*. *glabra* and *P*. *coriacea* shared an identical TLC R_f_ value with physciosporin in *P*. *granulata* and was pale or dark grey in daylight and became black and denser under UV (left and right panel, Fig 2B), we identified ‘spot c’ as physciosporin [21–23]. *P*. *faveolata* and *P*. *valdiviana* also possess physciosporin as their main compound (right panel, Fig 2B). The physciosporin used in this study was isolated from *P*. *coriacea* as the amounts of other physciosporin containing lichens were limited. Purity and structure of physciosporin was confirmed by HPLC and NMR analysis (Fig 2C; S1 File).

**Fig 3:**
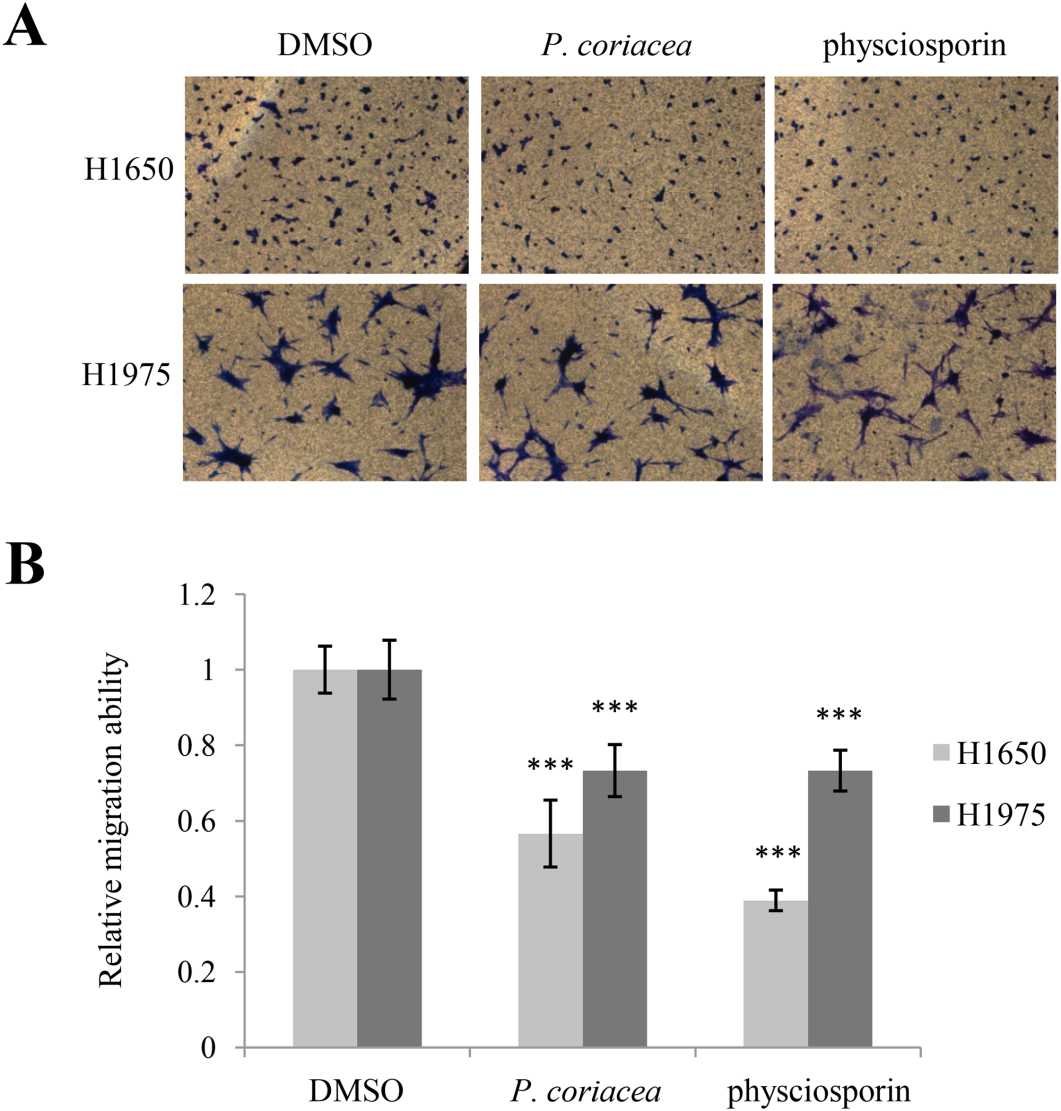
Acetone extract of *P*. *coriacea* and physciosporin inhibited lung cancer cell motility. (A-B) Invasion assays of H1650 and H1975 lung cancer cells treated with 5 μg/mL of acetone extract of *P*. *coriacea* or 5 μg/mL physciosporin (A), and quantitative analysis of invaded cell number in each treatment (B). Quantitative data were obtained from three independent experiments, n = 3. Data represent mean ± S.E.M. (standard error of the mean). ***p<0.001 compared to DMSO-treated A549 cells.

In order to check whether physciosporin is the active compound inhibiting A549 cell motility, physciosporin was tested in wound healing assays and invasion assays. Compared with the acetone extract of *P*. *coriacea*, physciosporin showed similar inhibitory activity against migration and invasion of A549 cells (Fig 2D–2G). In particular, physciosporin inhibited A549 cell invasion in a dose-dependent manner (Fig 2F and 2G). Interestingly, the inhibition of the purified compound was higher than that of crude extracts in the invasion assay but slightly lower in the migration assay (Fig 2E and 2G). Inhibitory activity of acetone extract of *P*. *coriaea* and physciosporin on lung cancer cell motility was also observed in H1650, H1975 cells (Fig 3). Cell viability was not altered at the given concentration of physciosporin (data not shown). Above all, these results demonstrated that physciosporin is an active lichen secondary metabolite from *P*. *coriacea* in the inhibition of lung cancer cell motility.

### Acetone extract of *P*. *coriacea* and physciosporin decreased the level of epithelial-mesenchymal transition marker

**Fig 4:**
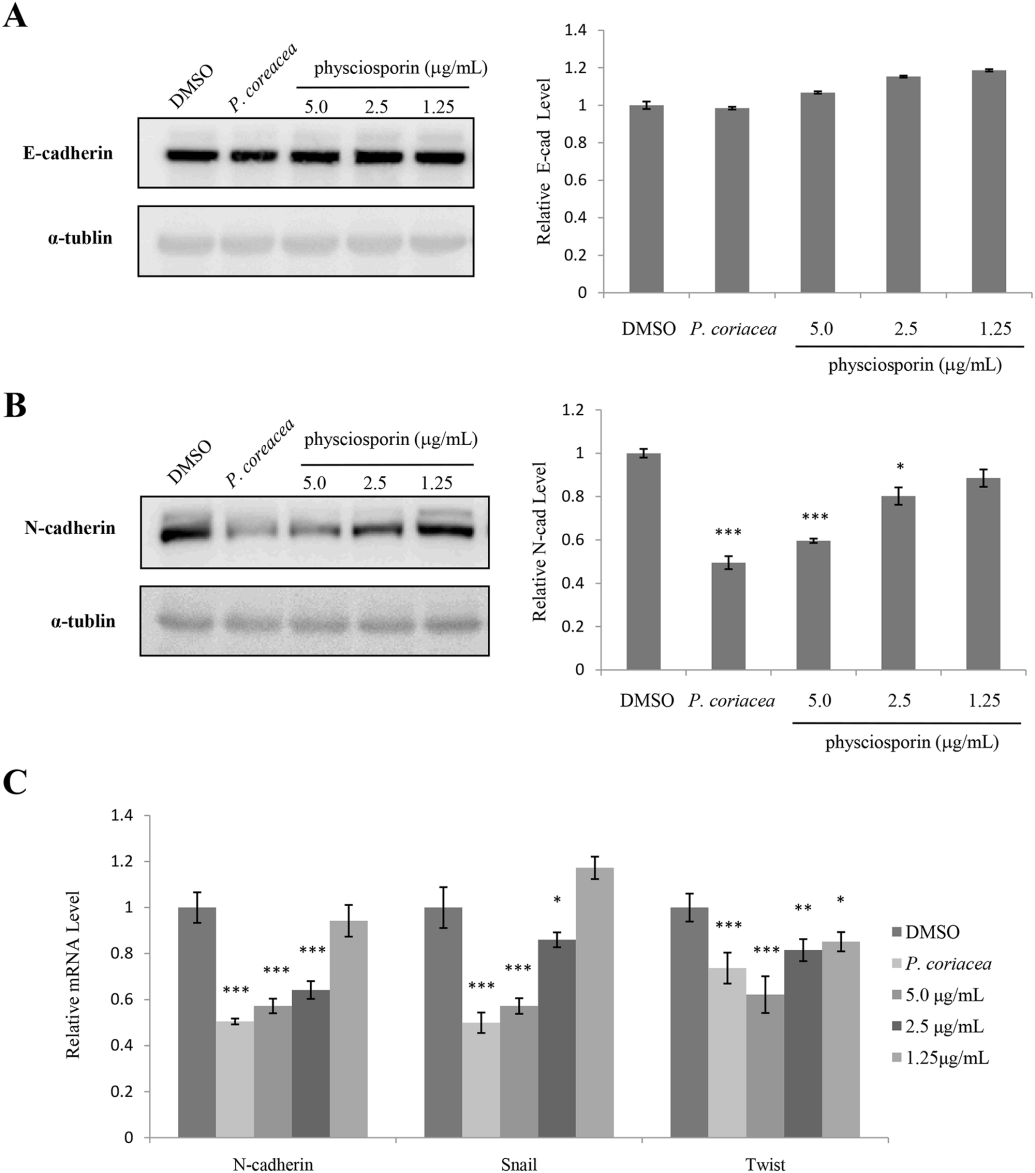
Acetone extract of *P*. *coriacea* and physciosporin decreased the level of epithelial-mesenchymal transition marker. (A-B) Western blot analysis of E-cadherin (A) and N-cadherin (B) in A549 cells treated with 5 μg/mL of acetone extracts of *P*. *coriacea* or physciosporin. (C) Quantitative analysis of the mRNA levels of N-cadherin, Snail and Twist in A549 cells treated with 5 μg/mL of acetone extracts of *P*. *coriacea* or physciosporin. Quantitative data were obtained from at least two independent experiments. Data represent mean ± S.E.M. (standard error of the mean). *p<0.05; **p<0.01; ***p<0.001 compared to DMSO-treated A549 cells.

To investigate the mechanism of action for the inhibitory activity of physciosporin against lung cancer cell motility, the effects of acetone extract of *P*. *coriacea* and physciosporin were examined in several signaling pathways. First, changes in the levels of epithelial-mesenchymal (EMT) marker were measured in treated cells (Fig 4). In our study, decreases in the expression of N-cadherin were prominent in A549 cells treated with acetone extract of *P*. *coriacea* or physciosporin at both the protein and mRNA levels (Fig 4B and 4C), while those of E-cadherin remained marginal (Fig 4A and data not shown for E-cadherin mRNA). In addition, the expressions of Snail and Twist were dose-dependently decreased by acetone extract of *P*. *coriacea* or physciosporin treatment (Fig 4C). Taken together, these results suggest that acetone extract of *P*. *coriacea* and physciosporin have inhibitory activity against lung cancer cell motility in part through inhibition of EMT.

### Acetone extract of *P*. *coriacea* and physciosporin suppressed KITENIN-mediated AP-1 activity and affected the expression of KAI1 and KITENIN

**Fig 5:**
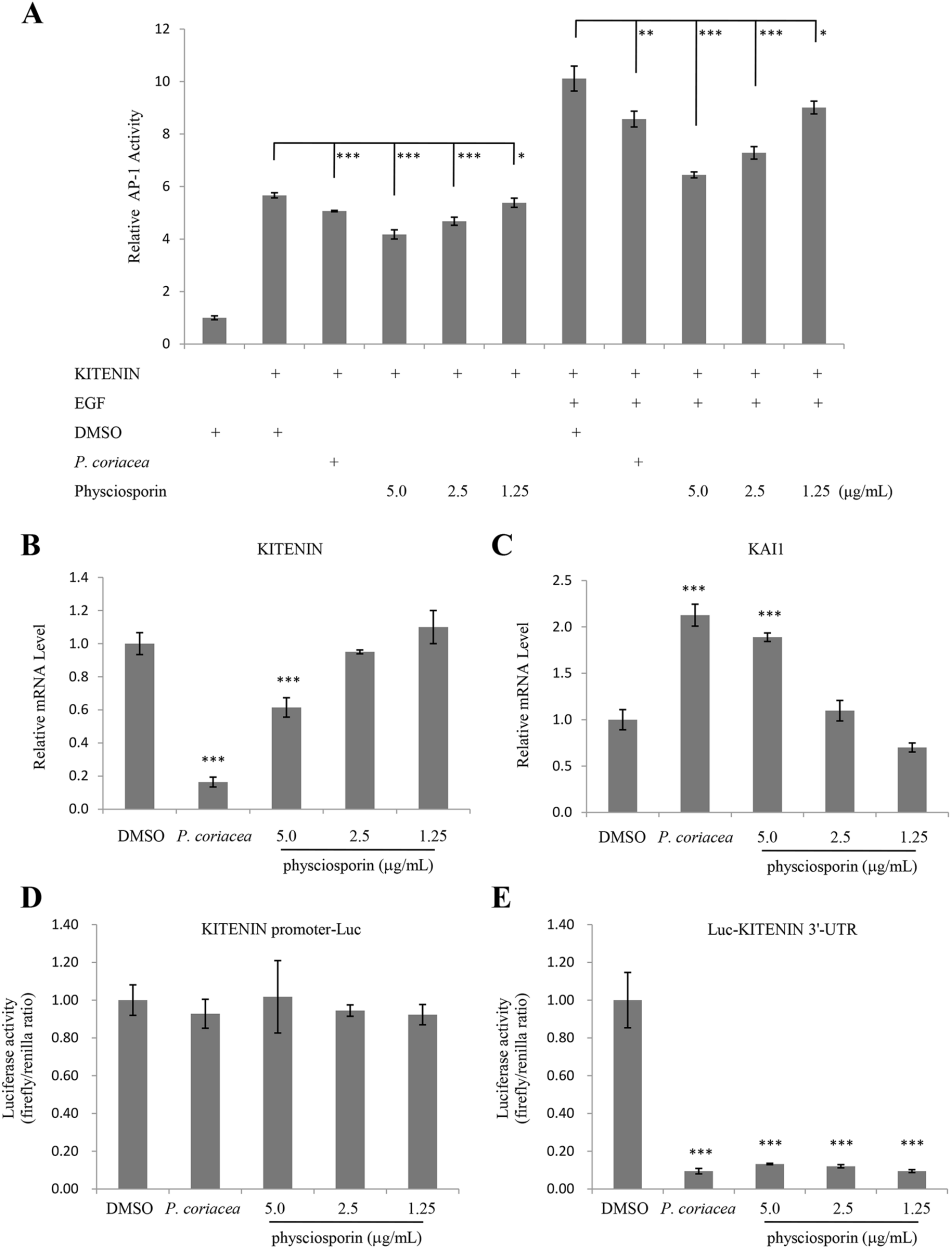
Acetone extract of *P*. *coriacea* and physciosporin suppressed KITENIN-mediated AP-1 activity and affected the expression of KAI1 and KITENIN. (A) AP-1 luciferase assay of HEK293T cells transfected with KITENIN and treated with 5 μg/mL of acetone extract of *P*. *coriacea* or physciosporin in the absence or presence of epidermal growth factor stimulation. (B-C) Quantitative analysis of the mRNA level of KITENIN (B) and KAI1 (C) in A549 cells treated with 5 μg/mL of acetone extract of *P*. *coriacea* or physciosporin. (D-E) KITENIN promoter (D) and 3’-UTR (E) luciferase assays of HEK293T cells treated with 5 μg/mL of acetone extract of *P*. *coriacea* or physciosporin. Quantitative data were obtained from at least three independent experiments. Data represent mean ± S.E.M. (standard error of the mean). *p<0.05; **p<0.01; ***p<0.001 compared to DMSO-treated cells.

Next, changes in transcriptional activities of β-catenin/LEF-1 (TOPFLASH), AP-1 (AP-1-luc), NF-κB (NF-κB-luc) and STAT (STAT-luc) were measured in treated cells. In a preliminary test, only AP-1 activity was decreased by acetone extract of *P*. *coriacea* or physciosporin treatment in a dose-dependent manner in the absence of co-activator (data not shown). In our previous study, we showed that KITENIN (KAI1 C-terminal interacting tetraspanin) promotes the tumorigenic potential of cancers, and epidermal growth factor (EGF) stimulates KITENIN-mediated AP-1 activation [24]. To further test the effects of acetone extract of *P*. *coriacea* or physciosporin on KITENIN-mediated AP-1 activity, AP-1 reporter assays were performed in cells co-transfected with KITENIN. As shown in Fig 5A, KITENIN-mediated AP-1 activity was decreased by acetone extract of *P*. *coriacea* or physciosporin treatment in a dose-dependent manner, both with and without EGF stimulation. In order to investigate how acetone extract of *P*. *coriacea* and physciosporin suppressed KITENIN-mediated AP1 activity, we tested KITENIN levels in treated cells. KITENIN mRNA levels were decreased by the treatment (Fig 5B). The metastatic suppressor gene, KAI1, is often down-regulated in metastatic tumor cells [25, 26]. As KITENIN has an inverse relationship with KAI1 or other metastasis suppressor genes [27], we tested the mRNA level of KAI1 in treated cells and found a similar inverse relationship between KITENIN and KAI1 (Fig 5C). Taken together, these results suggest that acetone extract of *P*. *coriacea* and physciosporin inhibit A549 cell motility in part through the suppression of KITENIN-mediated AP-1 activity by regulating KITENIN and KAI1 expressions. To check how mRNA level of KITENIN can be changed by the treatment, luciferase assays for KITENIN promoter and 3’-UTR were performed. As a result, the activity of KITENIN 3’-UTR was dramatically diminished by the treatment (Fig 5E) while that of KITENIN promoter remained constant (Fig 5D).

### Acetone extract of *P*. *coriacea* and physciosporin reduced RhoGTPase activity

**Fig 6:**
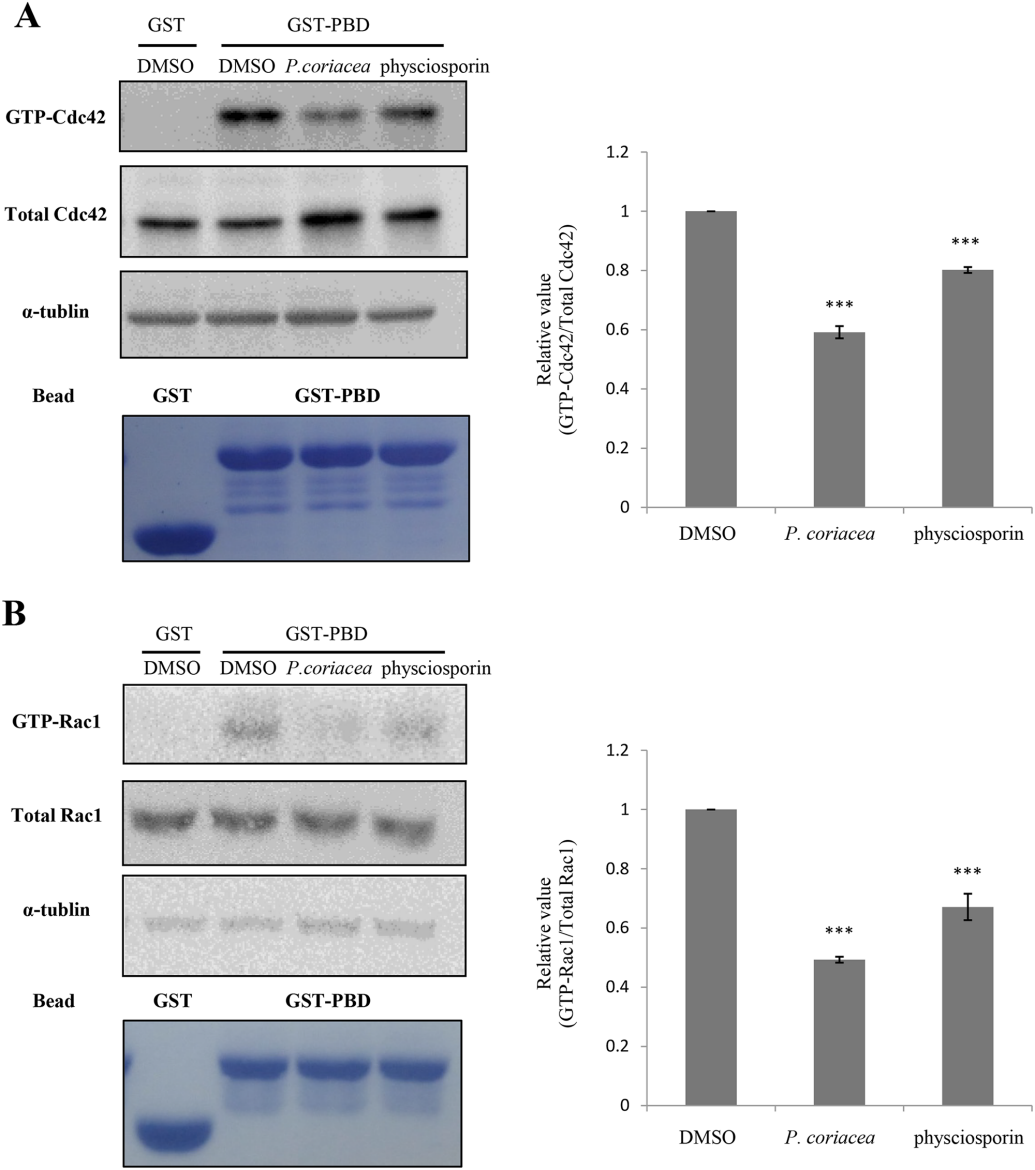
Acetone extract of *P*. *coriacea* and physciosporin reduced RhoGTPase activity. (A-B) The levels of GTP-bound Cdc42 (A) and Rac1 (B) were measured in A549 cells treated with 5 μg/mL of acetone extract of *P*. *coriacea* or physciosporin. GTP-Cdc42 and -Rac1 were measured using GST-PBD. The total amounts of Cdc42 and Rac1 were shown to measure relative activity. Quantitative data were obtained from three independent experiments, n = 3. Data represent the mean ± S.E.M. (standard error of the mean). ***p<0.001 compared to DMSO-treated A549 cells.

Next, changes in the activities of RhoGTPases were measured in treated cells (Fig 6). To investigate the effects of acetone extract of *P*. *coriacea* and physciosporin on the activities of Cdc42 and Rac1, GST pull-down assays were performed using GST-PBD (p21-binding domain). The results showed that the activities of Cdc42 and Rac1 were decreased by 20% and 33%, respectively, in the presence of physciosporin (Fig 6A and 6B). RhoA activity was also decreased by acetone extract of *P*. *coriacea* and physciosporin (data not shown). Taken together, these results suggest that acetone extract of *P*. *coriacea* and physciosporin inhibit A549 cell motility in part through the reduction of the RhoGTPase activity. The results demonstrate that the lichen secondary metabolite, physciosporin, inhibits cancer cell motility with specific mechanisms of action.

## Discussion

A lot of unique chemicals have been isolated from lichen used for pharmaceutical purpose. In this study, an effective chemical compound physciosporin has been isolated from Chile lichen *Pseudocyphellaria coriacea* for their inhibitory activity against lung cancer cell motility. Physciosporin, a chlorinated depsidone, was first isolated in 1977 from the lichen *Pseudocyphellaria physciospora* and identified as methyl 2-chloro-4-formyl-3,8-dihydroxy-l,6,9-trimethyl-11-oxo-11*H*-dibenzo [b,e] [l,4] dioxepin-7-carboxylate (methyl 5-chlorovirensate) [22], but their biological activity has not been reported to date. In this study, we found the following: 1) the acetone extract of *P*. *coriacea* and its subcomponent, physciosporin, inhibit motility of lung cancer cells; 2) the extract of *P*. *coriacea* and physciosporin suppress EMT; 3) the extract of *P*. *coriacea* and physciosporin decrease KITENIN-mediated AP-1 activity and affect the expression of KAI1 and KITENIN; and 4) the extract of *P*. *coriacea* and physciosporin reduce RhoGTPase activity.

EMT, an essential phenotypic conversion during embryonic development, tissue remodeling and wound healing, plays an indispensable role in tumor invasion and metastasis [28–30]. Increases in N-cadherin expression and decreases in E-cadherin level (called “cadherin switching”) are crucial in EMT and arise during cancer progression [31]. Especially, the level of N-cadherin seems to represent the mesenchymal phenotype of the cells and a group of transcription factors including Snail, Slug, ZEB and Twist has a crucial role in the control of EMT [32]. Snail, a zinc-finger transcription factor first identified in Drosophila, is a key EMT regulator [33]. Twist also has an important role in cancer metastasis. For example, inhibition of Twist expression by short-interfering RNAs affects metastatic vigor [34], and the regulation of Twist-1 may contribute to resistance to paclitaxel and antimicrotubule drugs in cancer patients [35]. Our result showed that the levels of N-cadherin, Snail, and Twist were decreased by acetone extract of *P*. *coriacea* and physciosporin treatment while those of E-cadherin remained marginal (Fig 4). Of note is that, in some case, changes in E-cadherin level are not essential for EMT [36] or metastatic activity of tumor [37].

KITENIN is a metastasis-enhancing gene. Recently, we identified that KITENIN/ErbB4-Dvl2-c-Jun axis is an unconventional EGFR-independent downstream signal of EGF and mediates the invasiveness and tumorigenesis of cancer cells [24]. In our results, KITENIN-mediated AP-1 activity was decreased by the extract of *P*. *coriacea* and physciosporin. Especially, the expression of KITENIN was decreased by the treatment and, for this, 3’-UTR activity of KITENIN was dramatically diminished (Fig 5). Few KITENIN-targeting microRNAs suppressing the migration and invasion of the cells via modulating KITENIN expression were reported and miR-27a, miR-30b, and miR-124 are among the examples [15]. Currently, we do not know whether physciosporin likely acts as microRNAs to suppress 3’-UTR activity of KITENIN. However, as unconventional KITENIN/ErbB4-mediated downstream signal of EGF plays one of the molecular basis for conferring resistance to anti-EGFR agents, the extract of *P*. *coriacea* and physciosporin may have potential beneficial activity in overcoming the limited clinical efficacy of anti-EGFR therapy.

Rho GTPases, members of the Ras superfamily of small GTPases such as Rac1, Cdc42, and RhoA, are adhesion and growth factor–activated molecular switches that play important roles in tumor development and progression [38]. Cdc42 is responsible for the fibroblastic morphology that leads to pulmonary metastasis of the cells via a mesenchymal mode of migration [39], and Rac1 drives the mesenchymal mode of migration [40]. Rac and Cdc42 also play a role in cell–cell adhesion in epithelial cells in addition to their effects on the actin cytoskeleton and motility. Our result showed that the activities of Rho GTPases were decreased by acetone extract of *P*. *coriacea* and physciosporin treatment (Fig 6). All of above results showed that physciosporin has anti-metastatic activity with molecular mechanisms of action, and further study is required to evaluate the potential anticancer activity of physiosporin in various *in vivo* cancer models.

## Supporting Information

S1 File ^1^H-NMR spectrum (Fig A) and ^13^C-NMR spectrum (Fig B) of physciosporin. (PDF)

## Abbreviations

DMSO: dimethylsulfoxide
EGF: Epidermal Growth Factor
HPLC: high-performance liquid chromatography
KITENIN: KAI1 C-terminal interacting tetraspanin
NMR: nuclear magnetic resonance
PBD: p21 binding domain
TLC: Thin Layer Chromatography
