# Supplementary material for: Lichen Secondary Metabolite, Physciosporin, Inhibits Lung Cancer Cell Motility
Source: PLoS One. 2015 Sep 15;10(9):e0137889. doi: 10.1371/journal.pone.0137889 (PMC4570789; doi:10.1371/journal.pone.0137889)
Supplement: S1 File — (PDF) [file pone.0137889.s001.pdf]

Supporting Information

Figure A. ^{1}H-NMR spectrum of physciosporin.

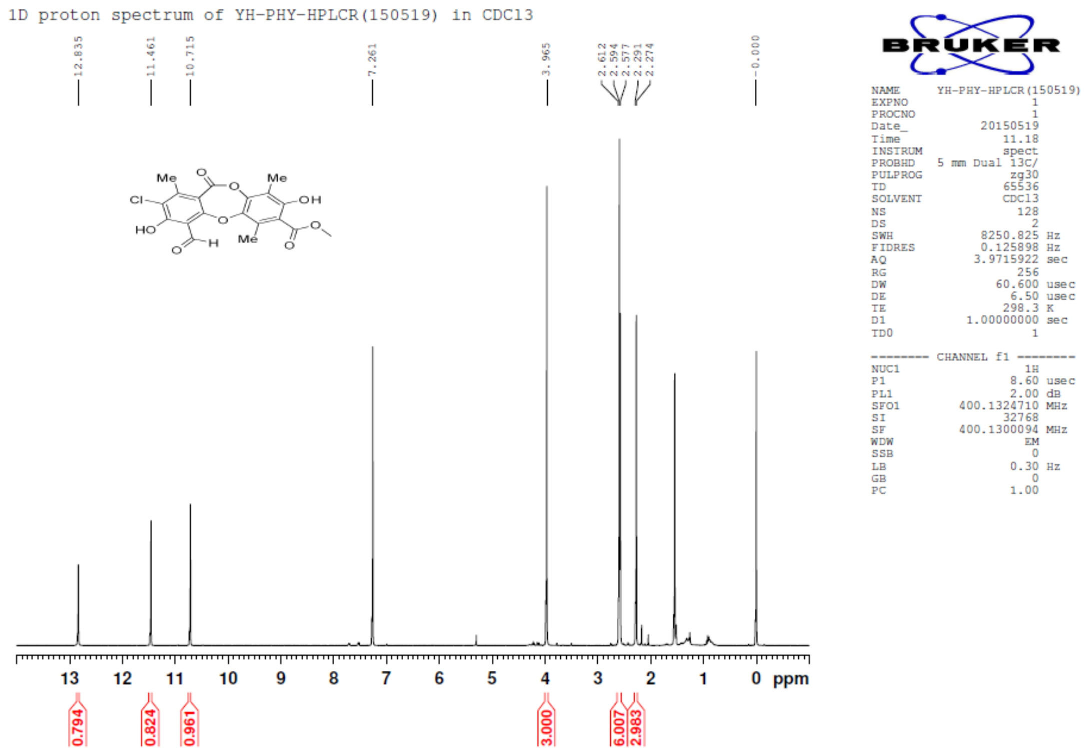

^{1}H-NMR (400 MHz, $CDCl_3$) δ 2.27 (s, 3H, CH3), 2.59 (s, 3H, CH3), 2.61 (s, 3H, CH3), 3.97 (s, 3H, COOCH3), 10.72 (s, 1H, CHO), 11.46 (s, 1H, OH), 12.83 (s, 1H, OH).

Figure B. ^{13}C -NMR spectrum of physciosporin.

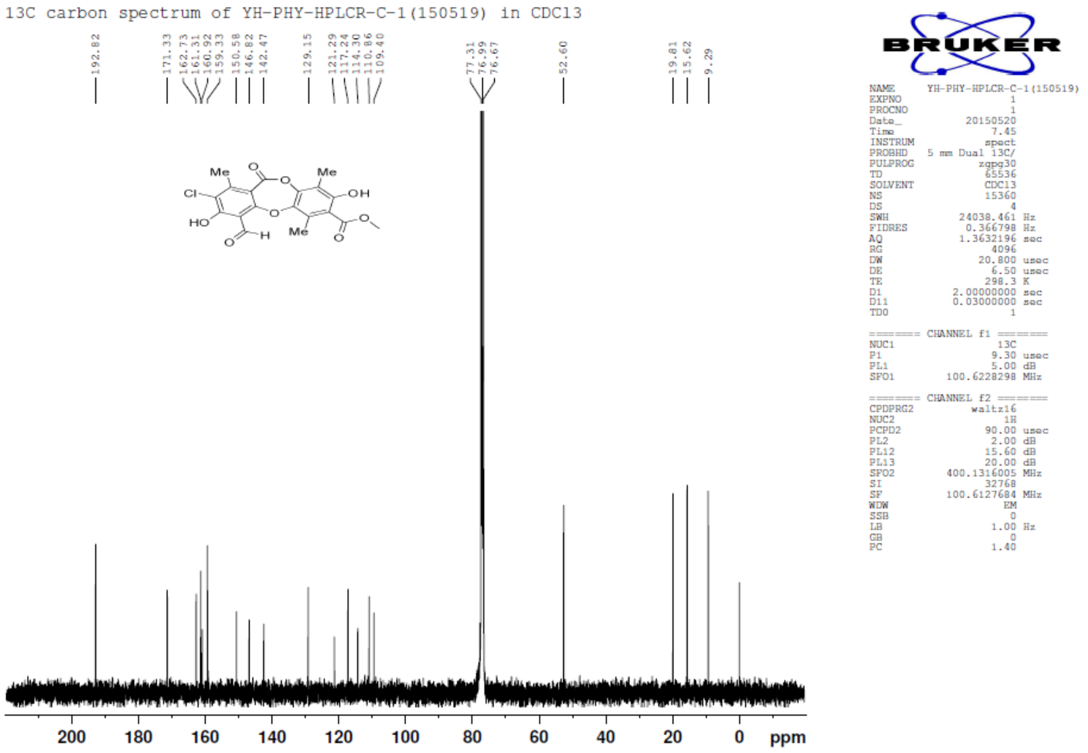

^{13}C-NMR (400MHz, $CDCl_3$) δ 15.62, 19.81, 52.60, 109.40, 110.86, 114.30, 117.24, 121.29, 129.15, 142.47, 146.82, 150.58, 159.33, 160.92, 161.31, 162.73, 171.33, 192.82.
